# *Borrelia* spp. in small mammals in Romania

**DOI:** 10.1186/s13071-019-3713-3

**Published:** 2019-09-24

**Authors:** Zsuzsa Kalmár, Attila Dávid Sándor, Ioana Adriana Matei, Angela Ionică, Gianluca D’Amico, Călin Mircea Gherman, Andrei Daniel Mihalca

**Affiliations:** 1 Department of Parasitology and Parasitic Diseases, Faculty of Veterinary Medicine, University of Agricultural Sciences and Veterinary Medicine Cluj-Napoca, Romania, Calea Mănăştur, No. 3-5, 400372 Cluj-Napoca, Romania; 2 Department of Microbiology, Immunology and Epidemiology, Faculty of Veterinary Medicine, Calea Mănăştur, No. 3-5, 400372 Cluj-Napoca, Romania; 3 “Regele Mihai I al României” Life Sciences Institute, University of Agricultural Sciences and Veterinary Medicine Cluj-Napoca, 3-5 Calea Mănăștur, 400372 Cluj-Napoca, Romania

**Keywords:** *Borrelia burgdorferi* (*s.l*.), *Borrelia miyamotoi*, *Ixodes ricinus*, Small mammals, Romania

## Abstract

**Background:**

Small mammals play an important role in the life-cycle of ticks and are reservoirs for several zoonotic pathogens. The aim of this study was to provide epidemiological data regarding the presence of *Borrelia* spp. in tissues of small mammals from Romania.

**Methods:**

We examined 401 individuals belonging to 11 small mammal species collected in Romania. Collections cover the largest effort to survey these reservoirs in the country. Tissue samples were analyzed by multiplex qPCR targeting the *ospA* gene of *Borrelia burgdorferi* (*s.l.*) and a part of the *flaB* gene of *B. miyamotoi*. Positive samples were further analysed by conventional PCR and sequenced.

**Results:**

The overall prevalence of infection with *Borrelia* spp. in small mammal tissues was 4.9%. The most commonly detected species were *B. afzelii*, followed by *B. garinii*/*B. bavariensis*, *B. miyamotoi* and *B. burgdorferi* (*s.s*.). To our knowledge, we report for the first time the detection of *Borrelia* spp. in *Crocidura leucodon* and *C*. *suaveolens*, and *B*. *miyamotoi* in the liver of *Myodes glareolus*.

**Conclusions:**

To our knowledge, our study evaluates for the first time the occurrence of *Borrelia* spp. in small mammals in Romania, contributing to a better knowledge of the distribution of these bacteria. This survey upgrades previous data on the spatial distribution of the pathogens and reveals the importance of animal surveillance regarding Lyme borreliosis and relapsing fever caused by *B. miyamotoi*.

## Background

Small mammals (Soricomorpha and Rodentia) are important reservoirs for many zoonotic tick-transmitted pathogens [1]. Several species of the genus *Ixodes* may serve as bridge vectors for *Borrelia* spp. that allow their circulation among different hosts, including small mammals [2] that are common hosts for the immature tick. Synanthropic micromammal species have been widely investigated because they act as reservoirs in the natural transmission cycles of *Borrelia* spp. [3]. Moreover, they are considered epidemiological markers in evaluating the distribution of certain tick species [4–7]. Although the infection rate with *Borrelia* spp. in these hosts is usually low, their local abundance may confer them an important epidemiological role [8].

Romania has a wide range of habitats colonized by species of small mammals and ticks. The small mammal-vector associations in Romania have been investigated by Mihalca et al. [4], who showed that the majority of the ticks on these vertebrates were *I. ricinus*, the most important tick parasitizing humans [7] and the only known vector for Lyme borreliosis in Europe. People living in rural areas in Romania are in close contact with the habitats preferred by small mammals and their ticks. This promotes a serious risk by tick-borne infectious agents that have a major impact on public health [9]. The aim of this study was to provide epidemiological data from a survey of the presence of *Borrelia* spp. in small mammals from Romania thus complementing existing surveys in ticks and other vertebrates.

## Methods

### Sampling

All animals were caught in Romania during 2010–2011, as previously described [4]. Each captured rodent was identified to the species level. A total of 401 small mammals of 11 species (*Apodemus agrarius*, *A. flavicollis*, *A. sylvaticus*, *A. uralensis*, *Micromys minutus*, *Microtus agrestis*, *M. arvalis*, *M. subterraneus*, *Myodes glareolus*, *Crocidura leucodon* and *C. suaveolens*) were collected from 7 counties of Romania. Tissue samples were collected from the animals: both the heart and liver from 393 animals (98%), only the heart from 2 animals (0.5%) and only the liver from 6 animals (1.5%).

### DNA extraction

Genetic material isolation was performed individually from tissues using a commercial DNA extraction kit (Isolate II Genomic DNA Kit; Bioline, London, UK) according to the manufacturer’s protocol. Extracted DNA was stored at − 20 °C for further analysis. For each extraction procedure, negative controls were used in order to identify possible cross-contamination.

### qPCR and sequencing

Multiplex quantitative polymerase chain reaction (qPCR) was used for evaluating the presence of *Borrelia* spp. in tissue samples. For *B. burgdorferi* (*s.l*.) we targeted the gene encoding the outer surface protein A (*ospA* gene) and for *B. miyamotoi* a part of the flagellin B (*flaB* gene). The qPCR was performed as previously described [10–12] in a CFX96 Touch™ Real-Time PCR detection system (Bio-Rad, London, UK) in a final reaction volume of 20 μl, using IQ Multiplex Powermix (Bio-Rad).

All the qPCR positive samples were amplified by conventional PCR and sequenced (Macrogen Inc., Amsterdam, Netherlands). Conventional PCR was performed as described by Szekeres et al. [8], targeting the glycerophosphodiester phosphodiesterase gene (*glpQ*) of *B. miyamotoi*. For *B. burgdorferi* (*s.l.*) the 5S-23S rDNA intergenic spacer region (IGS) and the outer surface protein A (*ospA*) gene were targeted [12]. Nucleotide sequences were compared with those available in GenBank using the Basic Local Alignment Search Tool. In each PCR reaction set, positive and two negative controls were included.

### Statistical analysis

Statistical analysis was performed by using Epi Info™ v.7.1.5 software. The frequency, infection prevalence and its 95% confidence interval were evaluated using a Chi-square independence test. A *P-*value of < 0.05 was considered statistically significant.

## Results

### Infection of small mammals with *Borrelia* spp

The overall prevalence of *Borrelia* spp. infection in micromammal tissues was 4.9% (95% CI: 3.2–7.5%). One out of two *M. agrestis* was infected (50%, 95% CI: 1.2–98.7%); infection prevalence was 14.2% in *C. suaveolens* (95% CI: 5.9–27.2%), 9.1% in *Mi. minutus* (95% CI: 0.2–41.2%), 6.2% in *My. glareolus* (95% CI: 0.7–20.8%), 5.6% in *M. arvalis* (95% CI: 1.1–15.6%), 4.7% in *C. leucodon* (95% CI: 0.1–23.8%), 4% in *A. flavicollis* (95% CI: 0.5–13.9%) and 3.4% in *A. agrarius* (95% CI: 0.7–9.7%). The infection prevalence was significantly different among micromammal species (*χ*^2^ = 23.6, *df* = 10, *P *< 0.01) suggesting a very different role in its intrinsic importance in the life-cycle of *Borrelia* spp. Three species, *A. sylvaticus*, *A. uralensis* and *M. subterraneus*, were not infected with *Borrelia* spp. (Table 1).

**Table 1:** The prevalence of *Borrelia* spp. in collected tissues

Host	Heart^a^	Liver^a^	Total^a^
*B. burgdorferi* (*s.l*.)			
*Apodemus agrarius*	2/83 (2.4)	3/86 (3.4)	3/87 (3.4)
*Apodemus flavicollis*	1/49 (2.0)	0/49 (0)	1/49 (2.0)
*Apodemus sylvaticus*	0/24 (0)	0/24 (0)	0/24 (0)
*Apodemus uralensis*	0/27 (0)	0/27 (0)	0/27 (0)
*Micromys minutus*	1/11 (9.0)	0/11 (0)	1/11 (9.1)
*Microtus agrestis*	1/2 (50.0)	1/2 (50.0)	1/2 (50.0)
*Microtus arvalis*	0/52 (0)	3/53 (5.6)	3/53 (5.6)
*Microtus subterraneus*	0/45 (0)	0/46 (0)	0/46 (0)
*Myodes glareolus*	1/32 (3.1)	0/32 (0)	1/32 (3.1)
*Crocidura leucodon*	1/21 (4.7)	0/21 (0)	1/21 (4.7)
*Crocidura suaveolens*	6/49 (12.2)	2/48 (4.1)	7/49 (14.2)
*B. miyamotoi*			
*Apodemus flavicollis*	0/49 (0)	1/49 (2.0)	1/49 (2.0)
*Myodes glareolus*	0/32 (0)	1/32 (3.1)	1/32 (3.1)
Total	13/395 (3.3)	11/399 (2.7)	20/401 (4.9)

^a^Positive/Tested (%)

### The diversity of *Borrelia* spp. in small mammal tissues

Three species of the *B. burgdorferi* (*s.l.*) complex (*B. afzelii*, *B. garinii*/*B. bavariensis* and *B. burgdorferi* (*s.s.*) and one species of the relapsing fever group (*B. miyamotoi*) were identified. The most frequently detected species was *B. afzelii* (70%; 95% CI: 45.7–88.1%) followed by *B. garinii*/*B. bavariensis*, *B. burgdorferi* (*s.s.*) and *B. miyamotoi* (10%, 95% CI: 1.2–31.7%) (Table 2).

**Table 2:** The frequency and prevalence of *Borrelia* spp. in tissues

Host	*N*	Prevalence (95% CI) (%)			
		BAF	BGA	BBSS	BMIYA
*Apodemus agrarius*	83	1.2 (0.03–6.24)	1.2/0.03–6.24	1.2 (0.03–6.24)	0 (0)
*Apodemus flavicollis*	49	2.0 (0.05–10.85)	0 (0)	0 (0)	2 (0.05–10.85)
*Apodemus sylvaticus*	24	0 (0)	0 (0)	0 (0)	0 (0)
*Apodemus uralensis*	27	0 (0)	0 (0)	0 (0)	0 (0)
*Crocidura leucodon*	21	4.8 (0.12–23.82)	0 (0)	0 (0)	0 (0)
*Crocidura suaveolens*	49	14.3 (5.94–27.24)	0 (0)	0 (0)	0 (0)
*Micromys minutus*	11	9.1 (0.23–41.28)	0 (0)	0 (0)	0 (0)
*Microtus agrestis*	2	50.0 (1.26–98.74)	0 (0)	0 (0)	0 (0)
*Microtus arvalis*	52	1.9 (0.05–10.07)	1.9 (0.05–10.07)	1.9 (0.05–10.07)	0 (0)
*Microtus subterraneus*	45	0 (0)	0 (0)	0 (0)	0 (0)
*Myodes glareolus*	32	3.1 (0.08–16.22)	0 (0)	0 (0)	3.1 (0.08–16.22)
Total	395	3.5 (2.1–5.8)	0.5 (0.1–1.8)	0.5 (0.14–1.8)	0.5 (0.1–1.8)

*Abbreviations*: *N*, total number of samples; BAF, *B. afzelii*; BGA, *B. garinii*; BBSS, *B. burgdorferi* (*s.s.*); BMIYA, *B. miyamotoi*

*Borrelia afzelii* was detected in 8 species: *A. agrarius* (GenBank: KY038873), *A. flavicollis* (KY038874), *C. leucodon* (KY123664), *C. suaveolens* (KY123665), *Mi. minutus* (KY123663), *M. agrestis* (KY123654), *M. arvalis* (KY123655) and *My. glareolus* (KY123656). *Borrelia garinii*/*B. bavariensis*, *B. burgdorferi* (*s.s.*) and *B. miyamotoi* were detected in single individuals of two species each: *B. garinii*/*B. bavariensis* in *A. agrarius* (KY123657), *B. garinii*/*B. bavariensis* in *M. arvalis* (KY123658), *B. burgdorferi* (*s.s*.) in *A. agrarius* (KY123659), *B. burgdorferi* (*s.s*.) in *M. arvalis* (KY123662), *B. miyamotoi* in *A. flavicollis* (KY123660) and *B. miyamotoi* in *My. glareolus* (KY123661).

### The infection rate in tissue samples

The overall infection rate was 3.2% in the heart and 2.7% in the liver, without significant difference between the tissue samples (*P* = 0.3) (Table 3). Four infections (20%; 95% CI: 5.7–43.7%) were detected in the heart and liver (two in *A. agrarius* and one each in *C. sueveolens* and *M. agrestis*), nine (45%; 95% CI: 23.1–68.5%) only in the heart (five in *C*. *suaveolens*, and one each in *A*. *flavicollis*, *C*. *leucodon*, *Mi*. *minutus* and *My*. *glareolus*) and seven (35%; 95% CI: 15.4–59.2%) only in the liver (three in *My*. *glareolus* and one each in *A*. *agrarius*, *A*. *flavicollis*, *C*. *suaveolens* and *My*. *glareolus*).

**Table 3:** The frequency of *Borrelia* spp. in the heart and liver of each animal

Host	Heart (+)						Liver (+)					
	*N*	*n*					*N*	*n*				
		BBSL	BAF	BGA	BBSS	BMIYA		BBSL	BAF	BGA	BBSS	BMIYA
*Apodemus agrarius*	83	2	0	1	1	0	86	3	1	1	1	0
*Apodemus flavicollis*	49	1	1	0	0	0	49	0	0	0	0	1
*Apodemus sylvaticus*	24	0	0	0	0	0	24	0	0	0	0	0
*Apodemus uralensis*	27	0	0	0	0	0	27	0	0	0	0	0
*Crocidura leucodon*	21	1	1	0	0	0	21	0	0	0	0	0
*Crocidura suaveolens*	49	6	6	0	0	0	48	2	2	0	0	0
*Micromys minutus*	11	1	1	0	0	0	11	0	0	0	0	0
*Microtus agrestis*	2	1	1	0	0	0	2	1	1	0	0	0
*Microtus arvalis*	52	0	0	0	0	0	53	3	1	1	1	0
*Microtus subterraneus*	45	0	0	0	0	0	46	0	0	0	0	0
*Myodes glareolus*	32	1	1	0	0	0	32	0	0	0	0	1
Total (%)	395	13 (3.2)	11 (2.7)	1 (0.2)	1 (0.2)	0 (0)	399	9 (2.2)	5 (1.2)	2 (0.5)	2 (0.5)	2 (0.5)

*Abbreviations*: *N*, total number of samples; *n*, number of positive samples; BBSL, *B. burgdorferi* (*s.l*.); BAF, *B. afzelii*; BGA, *B. garinii*; BMIYA, *B. miyamotoi*

From the total of 395 heart samples, 11 (95% CI: 1.6–4.9) were infected with *B. afzelii*, one (95% CI: 0.1–1.4) with *B. garinii*/*B. bavariensis* and *B. burgdorferi* (*s.s.*) In the liver, *B. afzelii* was detected in five samples (95% CI: 0.5–2.9), while *B. garinii*/*B. bavariensis*, *B. miyamotoi* and *B. burgdorferi* (*s.s.*) were each detected in two samples (95% CI: 0.1–1.8) (Table 2). None of the samples were co-infected.

Regarding the distribution of species by county, *B. afzelii* was found in Cluj (95% CI: 3.0–9.9), Constanţa (95% CI: 0.1–6.9) and Tulcea (95% CI: 0.1–26.0), *B. garinii*/*B. bavariensis* in Constanţa (95% CI: 0.1–6.9) and Bacău (95% CI: 0.8–90.6) counties, respectively *B. burgdorferi* (*s.s.*) in Constanţa (95% CI: 0.1–6.9) and Cluj (95% CI: 0.1–2.7) counties. *Borrelia miyamotoi* (95% CI: 0.1–3.5) was detected in *A. flavicollis* and *My. glareolus* trapped in Cluj (Table 4). *Borrelia* spp. DNA sequences detected in both tissues (heart and liver) in case of one specimen were 100% identical.

**Table 4:** The infection prevalence of *Borrelia* spp. in tissues of mammals captured in each county (see [3] for a review)

Host	No. of animals in each county^a^						
	Bacău	Cluj	Constanţa	Covasna	Harghita	Mureş	Tulcea
*B. burgdorferi* (*s.l*.)							
*Apodemus agrarius*	1/2 (50.0)	2/67 (2.9)	0/3 (0)	–	–	0/15 (0)	–
*Apodemus flavicollis*	0/1 (0)	1/17 (5.8)	–	–	–	0/26 (0)	0/5 (0)
*Apodemus sylvaticus*	–	0/8 (0)	0/10 (0)	0/1 (0)	–	0/4 (0)	0/1 (0)
*Apodemus uralensis*	–	–	0/19 (0)	–	0/2 (0)	0/2 (0)	0/4 (0)
*Crocidura leucodon*	–	1/18 (5.5)	–	–	–	0/2 (0)	0/1 (0)
*Crocidura suaveolens*	–	7/38 (18.4)	0/6 (0)	–	–	0/1 (0)	0/4 (0)
*Micromys minutus*	–	1/7 (14.2)	0/3 (0)	–	–	–	0/1 (0)
*Microtus agrestis*	–	–	–	–	–	–	1/2 (50.0)
*Microtus arvalis*	–	0/5 (0)	3/38/7.8	–	–	0/9 (0)	0/1 (0)
*Microtus subterraneus*	–	0/41 (0)	–	–	0/1 (0)	0/4 (0)	–
*Myodes glareolus*	–	1/6/16.6	–	–	–	0/26 (0)	–
*B. miyamotoi*							
*Apodemus flavicollis*	0/1 (0)	1/17 (5.8)	–	–	–	0/26 (0)	0/5 (0)
*Myodes glareolus*	–	1/6 (16.6)	–	–	–	0/26 (0)	–
Total	1/3 (33.3)	15/207 (7.2)	3/79 (3.8)	0/1 (0)	0/3 (0)	0/89 (0)	1/19 (5.2)

^a^Positive/Tested (%)

## Discussion

*Apodemus flavicollis*, *A. sylvaticus* and *My. glareolus* are the most common rodent species in Europe [8]. Their role in the circulation of *Borrelia* spp. is well acknowledged [9, 13, 14]. The present study shows that DNA of *Borrelia* spp. is prevalent in *A. agrarius*, *A. flavicollis*, *C. leucodon*, *C. suaveolens*, *Mi. minutus*, *M. agrestis*, *M. arvalis* and *My. glareolus* with a variable prevalence. Previous studies from European countries reported a slightly higher level of infection with *B*. *burgdorferi* (*s.l*.) in small mammal tissues. The prevalence reported in different countries is concurrent with the patchy distribution of *Borrelia* spp. For some species our data on prevalence was in line with other European countries, e.g. Croatia [15] and France [16] (from 7 to 7.5%) for *My. glareolus*.

The patterns of host association of different *Borrelia* spp. species are reported to differ remarkably [16]. The life-cycle of *B. afzelii* is dependent on multi-trophic interactions driven by the aggregation of ticks on rodents [17]. *Borrelia afzelii* has a large range of reservoirs, with small mammals commonly the most important reservoirs [14]. Bank voles and wood mice are considered preferred hosts for larvae of *I. ricinus* and may promote a high infection with *B*. *afzelii* in the resulting nymphs [18]. Moreover, *B. afzelii* has also been shown as the most common and widely distributed species of the Lyme group in questing [19–22] and engorged ticks [23–25] attached to humans, and in tissues of other vertebrates [26–29] in Romania. In this study, the majority of the small mammal species were infected with *B. afzelii* (70%). Similar prevalence rates were found in *A. agrarius* from Croatia (1.9%) and in *A. flavicollis* (1.5%). Studies from Hungary, Lithuania and Poland reported a slightly higher prevalence in *A. agrarius* and *A. flavicollis* [8, 30–32]. However, we consider that the inter-country comparison of prevalence is meaningless in this context, since the conditions of parasitism by ticks were different, as well as the environment and the period of the year for the surveys.

Small rodents have been found to be reservoirs of *B. garinii/B. bavariensis* and *B. valaisiana*. Overall, we found relatively low (10%) prevalence of *B. garinii*/*B. bavariensis* infection in small mammals. Different subtypes of *B. garinii* have been shown to be transmitted by birds. The distinct ecotype of *B. garinii* OspA serotype strains that actually corresponds to B. bavariensis utilizes rodents as reservoir hosts and has been associated with high pathogenicity in humans [33]. Our molecular detection protocol does not differentiate between *B. garinii* and *B. bavariensis*, thus further typing and/or multi-locus sequence analyses (MLSA) should be performed to delineate between *B. garinii* and *B. bavariensis* [34].

*Borrelia miyamotoi* is a tick-borne relapsing bacterium transmitted by *I. ricinus* in Europe. Reports from Hungary indicate the presence of *B. miyamotoi* in *A. flavicollis* (with infection prevalence of 4.8% in ticks, 0.3% in skin, 0.5% in the spleen) [8]. The presence of *B. miyamotoi* has been reported only in Asia (*A. argenteus*), North America (*Peromyscus leucopus*) and in Hungary (*My. glareolus*) [8, 33, 35, 36].

In the present study, shrews (*C. leucodon* and *C. suaveolens*) were found to have a higher infection rate (11.4%) in comparison to mice (3%) and voles (4.5%). Experimental studies on the reservoir role of the two insectivore species have not been performed so far. Here, we report the presence of *Borrelia* spp. in tissues of these species: *B. afzelii* was detected in the heart (*C. leucodon*, 1/21; *C. suaveolens*, 6/49) as well as in the liver (*C. suaveolens*, 1/48; liver and heart in a single case). As the number of the investigated shrews was relatively low, we are not able to conclude their relative contribution to the enzootic cycle of tick-borne pathogens.

The variety of natural habitats in Romania shapes a wide distribution of small mammals, the country being a natural focus for tick-borne rodent-associated zoonotic pathogens. These data, together with previous studies on the distribution of ticks and the prevalence of *Borrelia* spp. within them will contribute a general picture of the risk in the country.

## Conclusions

The pathogen-vector-reservoir interaction is fundamental to understand the epidemiology and prevent tick-borne diseases. Our analyses demonstrated that voles, mice and shrews are carriers of *Borrelia* spp. Consequently, the presence of different *Borrelia* species in the tissues of small mammals is an accurate marker of their circulation. These results are of importance for public health. To our knowledge, this is the first report of *Borrelia* spp. DNA in *C*. *leucodon* and *C*. *suaveolens*, and the presence of *B*. *miyamotoi* DNA in the liver of *My*. *glareolus*. Future studies are necessary to evaluate the contribution of shrews to the spread and maintenance of pathogens.

## Data Availability

The data supporting the conclusions of this article are included within the article. Representative sequences were submitted to the GenBank database under the accession numbers KY038873–KY038874 and KY123654–KY123665.

## Abbreviations

N: total number of samples
n: number of positive samples
BBSL: *B. burgdorferi* (*s.l*.)
BAF: *B. afzelii*
BGA: *B. garinii*
BMIYA: *B. miyamotoi*

## References

[1] Ostfeld RS, Levi T, Jolles AE, Martin LB, Hosseini PR, Keesing F (2014). Life history and demographic drivers of reservoir competence for three tick-borne zoonotic pathogens. PLoS ONE..

[2] Foley J, Piovia-Scott J (2014). Vector biodiversity did not associate with tick-borne pathogen prevalence in small mammal communities in northern and central California. Ticks Tick Borne Dis..

[3] Gern L, Humair PF, Gray J, Kahl O, Lane R, Stanek G (2002). Ecology of *Borrelia burgdorferi sensu lato* in Europe. Lyme borreliosis: biology, epidemiology and control.

[4] Mihalca AD, Dumitrache MO, Sándor AD, Magdaş C, Oltean M, Györke A (2012). Tick parasites of rodents in Romania: host preferences, community structure and geographical distribution. Parasit Vectors..

[5] Mihalca AD, Dumitrache MO, Magdaş C, Gherman CM, Domşa C, Mircean V (2012). Synopsis of the hard ticks (Acari: Ixodidae) of Romania with update on host associations and geographical distribution. Exp Appl Acarol.

[6] Hanincová K, Schäfer SM, Etti S, Sewell HS, Taragelova V, Ziak D (2003). Association of *Borrelia afzelii* with rodents in Europe. Parasitology..

[7] Briciu VT, Titilincu A, Ţăţulescu DF, Cârstina D, Lefkaditis M, Mihalca AD (2011). First survey on hard ticks (Ixodidae) collected from humans in Romania: possible risks for tick-borne diseases. Exp Appl Acarol.

[8] Szekeres S, Coipan EC, Rigó K, Majoros G, Jahfari S, Sprong H, Földvári G (2015). Eco-epidemiology of *Borrelia miyamotoi* and Lyme borreliosis spirochetes in a popular hunting and recreational forest area in Hungary. Parasit Vectors..

[9] Durden LA, Morand S, Krasnov BR, Poulin R (2006). Taxonomy, host associations, life cycles and vectorial importance of ticks parasitizing small mammals. Micromammals and macroparasites from evolutionary ecology to management.

[10] Heylen D, Tijsse E, Fonville M, Matthyse E, Sprong H (2013). Transmission dynamics of *Borrelia burgdorferi s.l.* in a bird tick community. Environ Microbiol..

[11] Hovius JW, De Wever B, Sohne M, Brouwer MC, Coumou J, Wagemakers A (2013). A case of meningoencephalitis by the relapsing fever spirochete *Borrelia miyamotoi* in Europe. Lancet..

[12] Coipan CE, van Duijvendijk GLA, Hofmeester TR, Takumi K, Sprong H (2018). The genetic diversity of *Borrelia afzelii* is not maintained by the diversity of the rodent hosts. Parasit Vectors..

[13] Tälleklint L, Jaenson TG (1994). Transmission of *Borrelia burgdorferi s.l.* from mammal reservoirs to the primary vector of Lyme borreliosis, *Ixodes ricinus* (Acari: Ixodidae), in Sweden. J Med Entomol..

[14] Gern L, Siegenthaler M, Hu CM, Leuba-Garcia S, Humair PF, Moret J (1994). *Borrelia burgdorferi* in rodents (*Apodemus flavicollis* and *A. sylvaticus*): duration and enhancement of infectivity for *Ixodes ricinus* ticks. Eur J Epidemiol..

[15] Tadin A, Tokarz R, Markotić A, Margaletić J, Turk N, Habuš J (2016). Molecular survey of zoonotic agents in rodents and other small mammals in Croatia. Am J Trop Med Hyg..

[16] Cosson JF, Michelet L, Chotte J, Le Naour E, Cote M, Devillers E (2014). Genetic characterization of the human relapsing fever spirochete *Borrelia miyamotoi* in vectors and animal reservoirs of Lyme disease spirochetes in France. Parasit Vectors..

[17] van Duijvendijk G, Coipan C, Wagemakers A, Fonville M, Ersöz J, Oei A (2016). Larvae of *Ixodes ricinus* transmit *Borrelia afzelii* and *B. miyamotoi* to vertebrate hosts. Parasit Vectors..

[18] Van Duijvendijk G, Sprong H, Takken W (2015). Multi-trophic interactions driving the transmission cycle of *Borrelia afzelii* between *Ixodes ricinus* and rodents: a review. Parasit Vectors..

[19] Kalmár Z, Mihalca AD, Dumitrache MO, Gherman CM, Magdaş C, Mircean V (2013). Geographical distribution and prevalence of *Borrelia burgdorferi* genospecies in questing *Ixodes ricinus* from Romania: a countrywide study. Ticks Tick Borne Dis..

[20] Coipan EC, Vladimirescu AF (2010). First report of Lyme disease spirochetes in ticks from Romania (Sibiu County). Exp Appl Acarol..

[21] Coipan EC, Vladimirescu AF (2011). *Ixodes ricinus* ticks (Acari: Ixodidae): vectors for Lyme disease spirochetes in Romania. Exp Appl Acarol..

[22] Ioniţă M, Mitrea IL, Pfister K, Hamel D, Silaghi C (2013). Molecular evidence for bacterial and protozoan pathogens in hard ticks from Romania. Vet Parasitol..

[23] Briciu VT, Meyer F, Sebah D, Tăţulescu DF, Coroiu G, Lupşe M (2014). Real-time PCR-based identification of *Borrelia burgdorferi sensu lato* species in ticks collected from humans in Romania. Ticks Tick Borne Dis..

[24] Briciu VT, Sebah D, Coroiu G, Lupşe M, Cârstina D, Ţăţulescu DF (2016). Immunohistochemistry and real-time PCR as diagnostic tools for detection of *Borrelia burgdorferi sensu lato* in ticks collected from humans. Exp Appl Acarol..

[25] Andersson M, Zaghdoudi-Allan N, Tamba P, Stefanache M, Chitimia L (2014). Co-infection with ‛*Candidatus* Neoehrlichia mikurensisʼ and *Borrelia afzelii* in an *Ixodes ricinus* tick that has bitten a human in Romania. Ticks Tick Borne Dis..

[26] Gherman C, Sándor AD, Kalmár Z, Marinov M, Mihalca AD (2012). First report of *Borrelia burgdorferi sensu lato* in two threatened carnivores: the marbled polecat, *Vormela peregusna* and European mink, *Mustela lutreola* (Mammalia: Mustelidae). BMC Vet Res..

[27] Paștiu AI, Matei IA, Mihalca AD, D’Amico G, Dumitrache MO, Kalmár Z (2012). Zoonotic pathogens associated with *Hyalomma aegyptium* in endangered tortoises: evidence for host-switching behaviour in ticks?. Parasit Vectors..

[28] Dumitrache MO, Paştiu AI, Kalmár Z, Mircean V, Sándor AD, Gherman CM (2013). Northern white-breasted hedgehogs *Erinaceus roumanicus* as hosts for ticks infected with *Borrelia burgdorferi sensu lato* and *Anaplasma phagocytophilum* in Romania. Ticks Tick Borne Dis..

[29] Dumitrache MO, Matei IA, Ionică MA, Kalmár Z, D’Amico G, Sikó-Barabási S (2015). Molecular detection of *Anaplasma phagocytophilum* and *Borrelia burgdorferi sensu lato* genospecies in red foxes (*Vulpes vulpes*) from Romania. Parasit Vectors..

[30] Paulauskas A, Ambrasiene D, Radzijevskaja J, Rosef O, Turcinaviciene J (2008). Diversity in prevalence and genospecies of *Borrelia burgdorferi sensu lato* in *Ixodes ricinus* ticks and rodents in Lithuania and Norway. Int J Med Microbiol.

[31] Gryczyńska A, Gortat T, Kowalec M (2018). Urban rodent reservoirs of *Borrelia* spp. in Warsaw, Poland. Epidemiol Infect..

[32] Michalik J, Skotarczak B, Skoracki M, Wodecka B, Sikora B, Hofman T (2005). *Borrelia burgdorferi sensu stricto* in yellow-necked mice and feeding *Ixodes ricinus* ticks in a forest habitat of west central Poland. J Med Entomol..

[33] Margos G, Wilske B, Sing A, Hizo-Teufel C, Cao WC, Chu C (2013). *Borrelia bavariensis* sp. nov. is widely distributed in Europe and Asia. Int J Syst Evol Microbiol..

[34] Margos G, Vollmer SA, Cornet M, Garnier M, Fingerle V, Wilske B (2009). A new *Borrelia* species defined by multilocus sequence analysis of housekeeping genes. Appl Environ Microbiol..

[35] Fukunaga M, Koreki Y (1995). The flagellin gene of *Borrelia miyamotoi* sp. nov. and its phylogenetic relationship among *Borrelia* species. FEMS Microbiol Lett..

[36] Bunikis J, Barbour AG (2005). Third *Borrelia* species in white-footed mice. Emerg Infect Dis..

